# Underutilization of Hepatitis C Virus Seropositive Donor Kidneys in the United States in the Current Opioid Epidemic and Direct-Acting Antiviral Era

**DOI:** 10.3390/diseases6030062

**Published:** 2018-07-10

**Authors:** Andrew A. Li, George Cholankeril, Xingxing S. Cheng, Jane C. Tan, Donghee Kim, Alice E. Toll, Satheesh Nair, Aijaz Ahmed

**Affiliations:** 1 Department of Medicine, Stanford University School of Medicine, Palo Alto, CA 94304, USA; andrewli@stanford.edu; 2 Division of Gastroenterology and Hepatology, Stanford University School of Medicine, Palo Alto, CA 94304, USA; georgetc@stanford.edu (G.C.); dhkimmd@stanford.edu (D.K.); 3 Division of Nephrology, Stanford University School of Medicine, Palo Alto, CA 94304, USA; xscheng@stanford.edu (X.S.C.); janetan@stanford.edu (J.C.T.); 4 United Network for Organ Sharing, Richmond, VA 23219, USA; Alice.Toll@unos.org; 5 Department of Transplant Surgery, Methodist University Hospital, University of Tennessee Health Science Center, Memphis, TN 38104, USA; snair@uthsc.edu

**Keywords:** hepatitis C virus, kidney transplantation, drug overdose, organ utilization, direct-acting antiviral agents

## Abstract

In recent years, the opioid epidemic and new hepatitis C virus (HCV) treatments have changed the landscape of organ procurement and allocation. We studied national trends in solid organ transplantation (2000–2016), focusing on graft utilization from HCV seropositive deceased donors in the pre-2014 (2000–2013) versus current (2014–2016) eras with a retrospective analysis of the United Network for Organ Sharing database. During the study period, HCV seropositive donors increased from 181 to 661 donors/year. The rate of HCV seropositive donor transplants doubled from 2014 to 2016. Heart and lung transplantation data were too few to analyze. A higher number of HCV seropositive livers were transplanted into HCV seropositive recipients during the current era: 374 versus 124 liver transplants/year. Utilization rates for liver transplantation reached parity between HCV seropositive and non-HCV donors. While the number of HCV seropositive kidneys transplanted to HCV seropositive recipients increased from 165.4 to 334.7 kidneys/year from the pre-2014 era to the current era, utilization rates for kidneys remained lower in HCV seropositive than in non-HCV donors. In conclusion, relative underutilization of kidneys from HCV seropositive versus non-HCV donors has persisted, in contrast to trends in liver transplantation.

## 1. Introduction

The demand for solid organ transplantation far exceeds the available supply of donor organs. Therefore, organ procurement organizations and transplant centers have begun to study protocols to expand the donor pool. One such strategy which has been in the forefront following the recent approval of direct-acting antiviral (DAA) agents is the use of organs from deceased donors with hepatitis C virus (HCV) infection. Second-generation DAA agents can achieve greater than 98% sustained virological response (SVR) rates in most HCV-infected patients, including in the post-transplant period [1,2,3,4]. 

In patients with end-stage renal disease (ESRD), kidney transplantation is considered the best replacement therapy. However, wait times for kidney transplants are over 3–5 years in most geographic areas of the United States (U.S.) [5]. Despite this imbalance between demand and supply of kidney donors, historic trends have demonstrated that up to 500 high-quality kidneys from HCV seropositive donors are discarded annually [6]. The discard rate for HCV seropositive kidney deceased donors is 2.6 times higher than for those from non-HCV donors [7]. Furthermore, only 29% of HCV seropositive recipients received kidneys procured from HCV seropositive donors, another missed opportunity to utilize these donors and reduce the organ shortage [8]. More recently, this practice has begun to change, with many transplant centers increasingly accepting kidneys from HCV seropositive donors for HCV seropositive recipients to increase the utilization of available organs and decrease waitlist times. Observational studies have clearly demonstrated that HCV seropositive recipients receiving HCV seropositive kidneys have a shorter waiting time [9,10,11,12]. 

Based on experience from the interferon era, there has been a historic reluctance to use kidneys from HCV seropositive donors due to a higher risk of mortality as compared to organs from non-HCV donors [9]. However, the introduction of DAA agents and their use in the peri-transplant setting has significantly changed this landscape. Early experience with interferon-free antiviral therapy in HCV-negative kidney transplant recipients of HCV viremic donors has demonstrated high SVR rates with the use of second-generation DAA agents in the post-transplant setting [4,13,14,15]. Combined with observational studies in ESRD patients demonstrating an overall benefit of kidney transplantation from HCV seropositive donors versus hemodialysis even in the pre-2014 era [16,17,18], these data support the argument to utilize organs from HCV seropositive donors for HCV-infected and even HCV-negative recipients.

This is of particular relevance with the recent increase in opioid use and a threefold increase in deaths related to drug overdose during the past decade in the U.S. [19], with an estimated 64,000 deaths attributed to drug overdose in 2016 [20]. Furthermore, it has been accompanied by an increase in the transmission rate of HCV infection in this particularly at-risk population with cases of HCV infection nearly tripling between 2011 and 2015 [20]. Therefore, the unfortunate deaths resulting from drug overdose in the young and relatively healthy adults in the U.S. have created an untapped source of donors with treatable HCV infection and an opportunity to expand the donor pool. A recent study examining drug overdose donors in kidney transplantation noted underutilization of HCV-infected donors [21]. We sought to further characterize changes in the utilization rates of organs from deceased donors with HCV seropositivity in solid organ transplantation, focusing on changes across the recent era and by recipient HCV status. Better understanding these data will help optimize organ utilization and maintain acceptable outcomes. 

## 2. Materials and Methods

We analyzed the U.S. national data from the Organ Procurement and Transplantation Network and the United Network for Organ Sharing (OPTN/UNOS) database. Data for kidney, liver, heart, and lung deceased donors procured, transplanted, and discarded in the U.S. from 1 January 2000 to 31 December 2016 among adult recipients (age 18 years and older) were analyzed. As nucleic acid testing (NAT) was not routinely recorded by OPTN/UNOS prior to 2015, HCV status was determined by serological antibody (Ab) testing. Utilization rate was defined as the number of organs transplanted per 100 organs procured on an annual basis. A subanalysis was performed to evaluate trends in kidney transplantation based on HCV seropositivity prior to and during the pre-2014 era. The current era was defined as January 1, 2014 and beyond, as second-generation DAA agents were first approved in the U.S. in October (Simeprevir) and November (Sofosbuvir) of 2013 [22]. The pre-2014 era was defined as prior to 1 October 2013. Donor demographic and clinical information analyzed among liver and kidney transplant recipients included age, ABO blood group status, Public Health Service (PHS) increased risk status, clinical infection, donor mechanism of death, liver donor risk index, and cold ischemic time. For recipients, age, Model for End-Stage Liver Disease (MELD) lab score at transplant, presence of hepatocellular carcinoma (HCC) diagnosis (ever), ascites at transplant, and encephalopathy and transplant were assessed. Comparisons among cohorts were reported as percentages for categorical variables and medians with interquartile range (IQR) were reported for continuous variables. When applicable, categorical variables were compared using a Chi-square test and continuous variables were compared using a Kruskal–Wallis test. Statistical significance was met with a *p*-value < 0.05. 

## 3. Results

**Figure 1:**
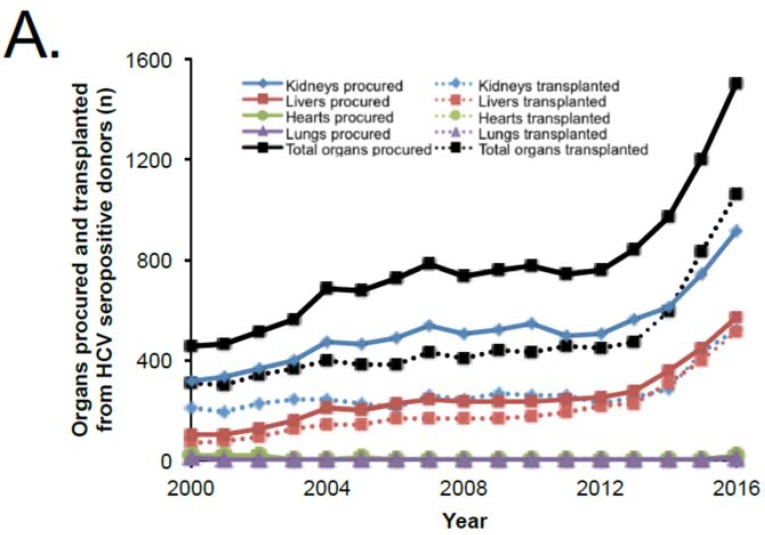

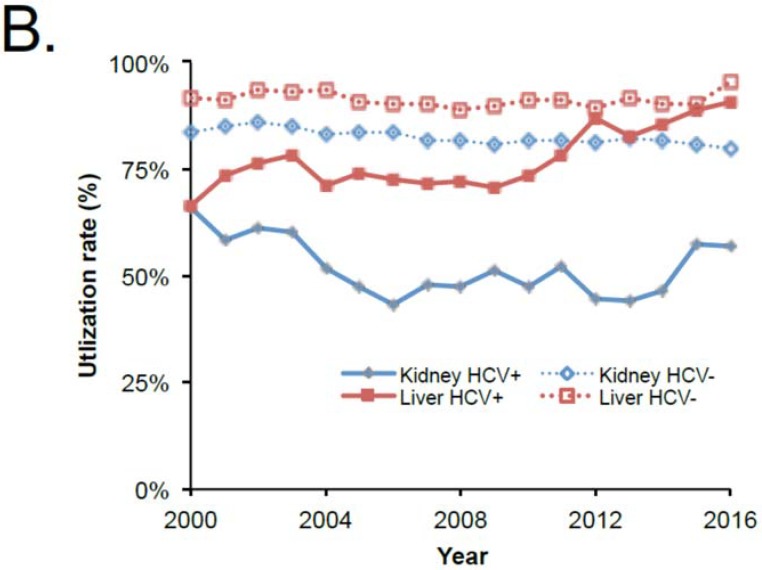
Trends in organ utilization from hepatitis C virus (HCV) seropositive donors. (**A**) Organs procured and transplanted from HCV seropositive donors by year; (**B**) Utilization rate (percentage organs procured that were transplanted) by HCV donor serostatus for liver and kidney transplantation. HCV+, HCV seropositive; HCV−, HCV seronegative.

Over the study period, the number of HCV seropositive organ donors increased from 181 to 661 per year (Table 1), corresponding to an increase from 3.0% of all organ donors in 2000 to 6.6% in 2016. Multiple organs can be procured from each donor; the rate of total solid organs (kidney, liver, heart, and lung) procured from HCV seropositive deceased donors increased from 452 organs per year in 2000 (2.1% of organs procured) to 1503 organs per year (4.3% of organs procured) in 2016. The incremental rise in the rate of HCV seropositive organs procured was most noticeable from 2013 to 2016 (Figure 1A). Along with the rise in the number of organs procured from HCV seropositive donors, a surge in the number of total solid organs transplanted was noted from 310 organs (1.7% of transplanted organs) in 2000 to 1058 organs (3.5% of transplanted organs) in 2016. The number of transplanted organs more than doubled from 2013 to 2016 following the approval of DAA agents in the U.S. On a per donor basis, the number of organs procured per donor has not appreciably changed over the study period (Table 1). However, there is a trend demonstrating a slightly higher number of organs transplanted per donor from HCV seropositive donors, increasing from 1.34 organs transplanted per donor in 2013 to 1.61 in 2016 (Table 1). However, the number of organs procured and transplanted per donor for HCV seropositive donors continues to be lower than that for their seronegative counterparts. 

In terms of liver transplantation, the rate of increase in livers transplanted from HCV seropositive donors has outpaced the increase in organs procured, with utilization rate rising from 66% to 91% over the study period (Figure 1B). Therefore, the utilization rate for HCV seropositive donor livers has reached parity with non-HCV donors, with the number of livers transplanted from HCV seropositive donors more than doubling from 2013 to 2016. 

**Figure 2:**
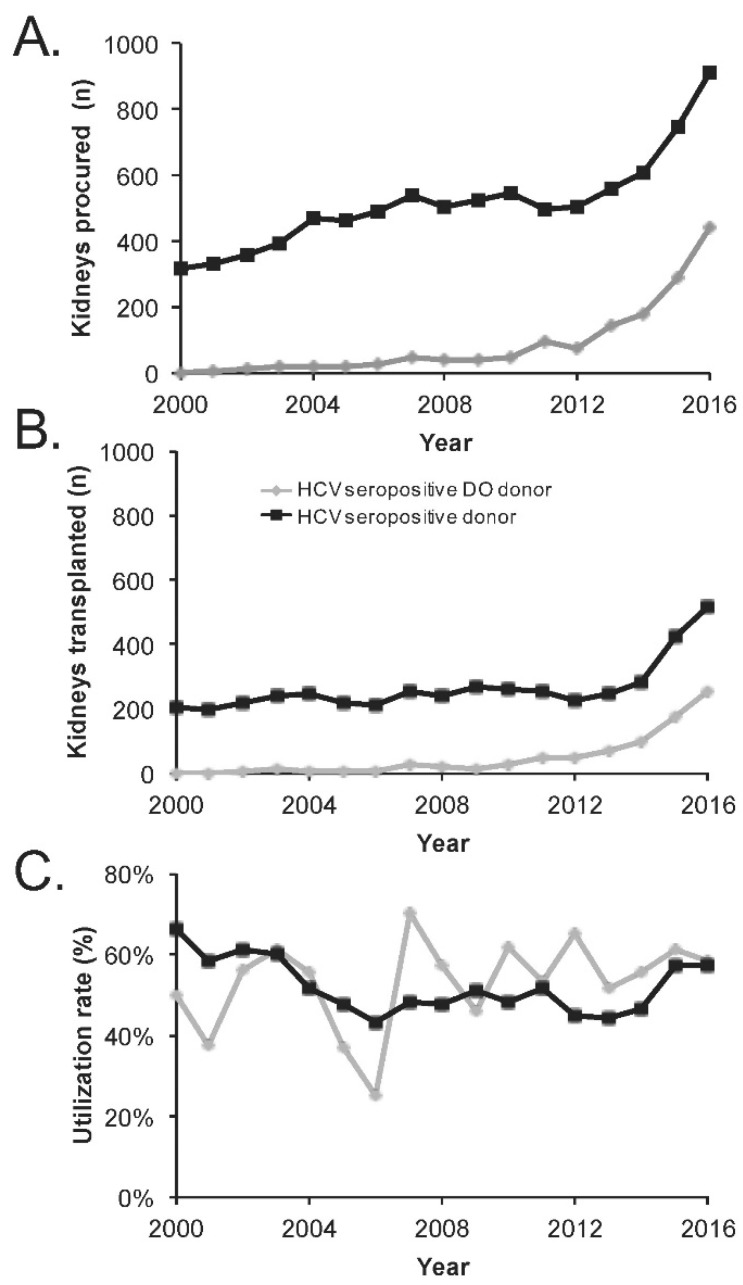
Trends in kidney utilization from HCV seropositive and drug overdose donors. (**A**) Kidneys procured from all HCV seropositive donors and from HCV seropositive donors with drug overdose as the mechanism of death; (**B**) Kidneys transplanted from the same populations; (**C**) Utilization rate of kidneys procured from the same populations. DO = Drug Overdose as the mechanism of death.

This higher rate of utilization in livers is contrasting with patterns in utilization of kidneys from HCV seropositive donors. Although the absolute number of kidney transplants from HCV seropositive donors has increased over twofold from 247 kidneys in 2013 to 520 kidneys in 2016, this has not kept pace with the number of available kidneys. When compared to kidneys from HCV seronegative donors, kidneys from HCV seropositive donors have been persistently utilized at a lower rate (57% HCV seropositive versus 80% HCV seronegative in 2016) in contrast to the recent patterns realized in liver transplants. Overall, the utilization rate of non-HCV organs has remained essentially unchanged during the study period for both kidneys and livers. In heart and lung transplants, the numbers of organs procured and transplanted are too small to derive meaningful trends, but, notably, there were 17 hearts and 6 lungs transplanted from HCV seropositive donors in 2016—an increase from the 0 to 4 transplants per year in the three preceding years. Focusing on kidneys, a high percentage of kidneys from HCV seropositive donors are procured from drug overdose fatalities—approximately 49% in 2016 (Figure 2A)—with an increase from 142 kidneys in 2012 to 442 kidneys in 2016. Of these, 73 kidneys were transplanted in 2013 and 258 were transplanted in 2016, reflecting a utilization rate that was largely unchanged in the 51–62% range (Figure 2B). A similar utilization rate is noted in HCV seropositive drug overdose donors compared to all HCV seropositive donors over the study period. The vast majority of the increase in HCV seropositive kidneys procured is driven by the increase in drug overdose kidneys, each year accounting for greater than 75% of the annual increase in HCV seropositive donors from 2012 to 2016.

As several changes occurred near 2014 with the introduction of second-generation DAAs and a surge in drug-overdose-related deaths, we compared the transplantation trends for organs from HCV seropositive donors in the pre-2014 (2000 to late 2013) versus current (2014–2016) eras based on recipient HCV serostatus (Table 2). A higher number of livers from HCV seropositive donors were transplanted into HCV seropositive recipients during the pre-2014 era with 374 liver transplants per year (59.6 per 1000 transplants) in the current era as compared to 124 liver transplants per year (25.3 per 1000 transplants) in the pre-2014 era. The current era was also associated with an increase in the absolute number of livers from HCV seropositive donors into HCV seronegative recipients from 20.3 to 25.0 liver transplants per year, but the frequency was unchanged (4.1 versus 4.0 per 1000 transplants in the pre-2014 versus current era). In kidney transplantation, the number of kidneys from HCV seropositive donors transplanted to HCV seropositive recipients increased from 165.4 to 334.7 kidneys per year (17.5 pre-2014 to 28.2 current per 1000 transplants), but the number of such kidneys transplanted into seronegative recipients decreased from 44.1 to 34.3 per year (4.7 pre-2014 to 2.9 current per 1000 transplants) in the current versus pre-2014 era.

Demographics and clinical characteristics of HCV seropositive kidney donors and their recipients are shown in Table S1. As compared with their seronegative counterparts, HCV seropositive donors were younger (33 versus 40 years old, *p* < 0.0001), more likely to be white and male, and with a higher CDC HIV risk. Nearly a third of HCV seropositive donors died from drug overdose during the study period. Recipients of HCV seropositive organs were older (59 years vs. 56 years, *p* < 0.0001) and more likely to be male (80.2% vs. 59.9%, *p* < 0.0001) and black (62.3% vs. 32.7%, *p* < 0.0001). A subanalysis focused on HCV seropositive donors stratified by pre-2014 versus current era (Table 3 and Table 4). During the current era, the average donor age was significantly lower for both kidney and liver transplants with a higher frequency of drug intoxication as the cause of donor death. An increased tolerance and acceptance for using high-risk donors labeled as “donor clinical infection” and/or “Public Health Service (PHS) increased risk” for both liver and kidney transplants was noted. In liver transplant recipients, the current era was associated with significantly lower frequency of ascites (78% vs. 70%, *p* < 0.0001) and hepatic encephalopathy (67% vs. 55%, *p* < 0.0001) but without any significant difference in MELD at time of transplant. Notably, the Kidney Donor Profile Index (KDPI) for kidney [23] and the Donor Risk Index (DRI) for liver [24], models for estimating the risk of graft failure, are also significantly lower in the current era, indicating lower predicted risks of graft failure, likely reflecting the younger age of these donors.

## 4. Discussion

In our analysis of historical trends of organs from HCV seropositive donors, we find that utilization patterns vary by organ and era. In the current era, there has been a significant increase in the number of organs procured from HCV seropositive donors. In liver transplant recipients, organs from HCV seropositive deceased donors are now being utilized at similar rates as those from donors without evidence of HCV infection. On the contrary, the utilization rate of kidneys from HCV seropositive deceased donors has been largely unchanged and is lower compared to non-HCV deceased donors, although there has been an increase in the absolute number of kidney transplants from the HCV seropositive pool. Interestingly, while there has been an increase in the annual rate of kidney transplants from HCV seropositive donors to HCV seropositive recipients, the rate of transplantation of such kidneys into seronegative recipients has decreased in the current era. This is in contrast to trends observed in liver transplant programs in the U.S., acknowledging that given the higher rate of HCV infection in liver recipients versus kidney recipients these rates may not be expected to reach parity. However, combined with the surge in the number of HCV seropositive deceased donors related to drug overdose and the introduction of DAAs, this presents a unique set of circumstances to potentially expand the donor pool which warrants further examination.

An important distinction should be made between HCV seropositivity (antibody status) and HCV RNA positivity (active viremia) by nucleic acid testing (NAT). According to OPTN/UNOS, approximately two-thirds of HCV antibody (Ab)-positive donors are also NAT positive and, thus, with active viremia [25,26]. The OPTN/UNOS registry began collecting data on NAT status in 2015; thus, longer-term historical trends were not available. However, recent studies have reported results with similar trends demonstrating underutilization in HCV Ab- or NAT-positive donor organs compared with their HCV-negative counterparts [26]. While livers from HCV Ab- and NAT-positive donors were used at comparable rates in 2015 and 2016, other organs were utilized at lower rates from HCV seropositive donors. Our study demonstrates that the relative underutilization of kidneys from HCV seropositive versus HCV seronegative deceased donors has persisted despite the availability of DAA agents and lower KDPI index. 

Our analysis focuses on trends in the utilization rate of deceased donors based on HCV serostatus with or without drug overdose since the introduction of DAA agents and the current opioid epidemic. As several concurrent changes transpired during the 2013–2016 timeframe, these trends and the relatively lower utilization of kidneys cannot be ascribed to an isolated factor. One possible factor is a reluctance to use kidneys from HCV seropositive donors given the historically comparatively poorer outcomes from the interferon era and/or concerns about lower graft quality; however, a recent study of HCV Ab-positive, NAT-negative kidney transplants compared with KPDI-matched Ab-negative, NAT-negative controls had similar graft survival [27]. Recipients of these Ab-positive, NAT-negative kidneys also had shorter wait list times and tended to be HCV Ab positive. Other reasons include a possible greater number of speculatively procured kidneys. For example, there may be a lower utilization rate of speculatively procured kidneys from older HCV seropositive donors whose livers are being transplanted, though the younger age of most drug-overdose donors driving the increase in HCV seropositive donors argues against this. Additionally, for HCV seropositive donors who die of drug overdose and experience prolonged anoxia prior to presentation, livers may be utilized while speculatively procured kidneys could be less viable and, thus, less utilized; this remains an area that needs further investigation. 

When considering the option of transplantation utilizing organs from HCV-infected, namely, NAT-positive donors, the benefits of earlier transplantation must be weighed against the risks of HCV infection; this risk may be somewhat mitigated in the current era. The risk of negatively impacting the outcomes is lower in theory with a rise in the proportion of younger and relatively healthy HCV seropositive deceased donors from drug overdose. Furthermore, prompt treatment with DAA-based antiviral therapy in the peri-transplant period provides added assurance for a favorable outcome. Most such transplantations thus far have been into HCV seropositive recipients, and similar graft survival has been seen in kidneys and livers [27,28]. Transplantation of HCV-infected kidneys into HCV seronegative recipients currently occurs nearly exclusively in ongoing clinical trials, but early experience, such as in the EXPANDER and THINKER trials, indicates successful treatment of HCV in the post-transplant setting with SVR rates comparable to nontransplant populations [3,4,15]. Additionally, not all HCV Ab-positive donors are NAT positive; a subset of those who are HCV Ab positive and NAT negative due to prior infection with spontaneous clearance or prior HCV infection with SVR following antiviral therapy have a very low risk of viral transmission. Patients should be appropriately counseled regarding this risk, as well as the risk for other potentially communicable diseases such as hepatitis B virus and human immunodeficiency virus. Organs from this subset of HCV seropositive donors are relatively lower risk and their usage should be more strongly considered given the currently available outcomes data and relatively underutilized organs.

Our study is limited by the retrospective design and its use of HCV Ab status rather than NAT status, but, as noted above, NAT status was not captured by the OPTN/UNOS database prior to 2015. This use of NAT status may also have also contributed to an era effect with the more refined use of HCV seropositive, NAT-negative donor organs. Additionally, although DAA agents were available after late 2013, data on their use in either donors or recipients were not available. While donation rates for HCV seropositive donors may also be confounded by lower overall rates of organ procurement, this would presumably make the above estimates of organ utilization higher than they would be otherwise and argue for an even larger potential donor pool. Further work will need to be performed to assess outcomes in HCV seropositive organs as KPDI and other predictive models are of more limited utility in this setting due to being significantly influenced by the age of donors. Finally, we note that emerging data from prospective trials [3,4,15,29] and post-transplant patient survival data demonstrate encouraging short-term outcomes, but longer-term outcomes from HCV seropositive organs will need to be studied.

The advantages of utilizing HCV seropositive organs include an expansion of the donor pool; younger and better quality organs due to lack of co-morbid medical conditions; shorter wait times and transplant surgery at a relatively stable clinical status; and the associated cost savings [5]. However, several unanswered questions remain about how to best select and match donors with recipients, the timing of post-transplant DAA therapy, and longer-term outcomes despite promising early results. Our data highlight trends in the use of organs from HCV seropositive donors and an opportunity to expand the supply of suitable kidneys for transplantation. 

## Figures and Tables

**Table 1:** Organs procured and transplanted by HCV donor serostatus.

	HCV Seropositive Donors					HCV Seronegative Donors				
	Donors	Organs Procured		Organs Transplanted		Donors	Organs Procured		Organs Transplanted	
Year	*N*	*N*	Per Donor	*N*	Per Donor	*N*	*N*	Per Donor	*N*	Per Donor
2000	181	460	2.54	316	1.75	5804	21,039	3.62	18,710	3.22
2001	197	473	2.4	301	1.53	5883	21,451	3.65	19,054	3.24
2002	213	519	2.44	343	1.61	5977	21,895	3.66	19,763	3.31
2003	252	562	2.23	369	1.46	6205	22,337	3.6	20,039	3.23
2004	301	690	2.29	404	1.34	6849	24,453	3.57	21,562	3.15
2005	285	679	2.38	379	1.33	7308	26,152	3.58	22,980	3.14
2006	322	726	2.25	383	1.19	7695	27,512	3.58	24,121	3.13
2007	352	780	2.22	433	1.23	7733	27,525	3.56	23,841	3.08
2008	335	741	2.21	411	1.23	7654	27,135	3.55	23,568	3.08
2009	348	773	2.22	448	1.29	7674	27,523	3.59	23,841	3.11
2010	331	783	2.37	439	1.33	7612	27,681	3.64	24,226	3.18
2011	320	751	2.35	458	1.43	7806	28,095	3.6	24,574	3.15
2012	335	765	2.28	453	1.35	7808	27,837	3.57	24,172	3.1
2013	361	843	2.34	482	1.34	7907	28,560	3.61	25,031	3.17
2014	436	976	2.24	601	1.38	8160	29,182	3.58	25,509	3.13
2015	535	1202	2.25	832	1.56	8544	30,715	3.59	26,708	3.13
2016	661	1506	2.28	1061	1.61	9309	33,854	3.64	29,436	3.16

**Table 2:** Comparison of average annual rates of transplantation (annual number of transplants per 1000 transplants) of organs from HCV seropositive donors by recipient HCV serostatus in the pre-2014 and current eras.

	HCV Seropositive Donor HCV Seropositive Recipient		HCV Seropositive Donor HCV Seronegative Recipient	
	Pre-2014 Era	Current Era	Pre-2014 Era	Current Era
Heart	0.67	0.55	3.01	2.23
Lung	0.18	0.00	0.54	0.40
Liver	25.27	59.62	4.09	3.99
Kidney	17.50	28.23	4.67	2.89

**Table 3:** Kidney demographics and clinical characteristics from HCV seropositive donors by era.

	Pre-2014 Era (*N* = 2274)	Current Era (*N* = 1004)	*p* Value
**Donor age (years)**	44.0 (34.0–49.0)	32.0 (26.0–39.0)	<0.001
**Donor gender**			0.860
Female	800 (35.2%)	350 (34.9%)	
Male	1474 (64.8%)	654 (65.1%)	
**Donor ethnicity**			<0.001
White	1681 (73.9%)	847 (84.4%)	
Black	319 (14.0%)	54 (5.4%)	
Hispanic	247 (10.9%)	91 (9.1%)	
**Donor ABO**			0.503
A	701 (30.8%)	327 (32.6%)	
Ab	15 (0.7%)	10 (1.0%)	
B	281 (12.4%)	115 (11.5%)	
O	1277 (56.2%)	552 (55.0%)	
**Donor PHS increased risk**	666 (39.2%)	781 (77.8%)	<0.001
**Donor clinical infection**	1022 (44.9%)	724 (72.2%)	<0.001
**Donor mechanism of death**			<0.001
Drug intoxication	228 (10.0%)	433 (43.1%)	
Asphyxiation	60 (2.6%)	73 (7.3%)	
Cardiovascular	177 (7.8%)	107 (10.7%)	
Gunshot wound	261 (11.5%)	83 (8.3%)	
Blunt injury	546 (24.0%)	171 (17.0%)	
ICH/stroke	905 (39.8%)	108 (10.8%)	
Death from natural causes	35 (1.5%)	11 (1.1%)	
None of the above	39 (1.7%)	10 (1.0%)	
**KDPI (ref. Population = 2016)**	68.0 (50.0–84.0)	49.0 (37.0–63.0)	<0.001
**Cold ischemic time (hours)**	19.0 (13.4–24.4)	16.6 (11.0–23.0)	<0.001
**Recipient age**	55.0 (49.0–59.0)	60.0 (56.0–64.0)	<0.001
Recipient dialysis prior	2077 (91.3%)	861 (85.8%)	<0.001

PHS, Public Health Service; ICH, intracranial hemorrhage; KDPI, Kidney Donor Profile Index.

**Table 4:** Liver demographics and clinical characteristics from HCV seropositive donors by era.

	Pre-2014 Era (*N* = 1724)	Current Era (*N* = 1122)	*p* Value
**Donor age (years)**	44.0 (32.0–51.0)	35.0 (28.0–47.0)	<0.001
**Donor gender**			0.586
Female	657 (38.1%)	439 (39.1%)	
Male	1067 (61.9%)	683 (60.9%)	
**Donor ethnicity**			<0.001
White	1200 (69.6%)	910 (81.1%)	
Black	344 (20.0%)	120 (10.7%)	
Hispanic	156 (9.0%)	74 (6.6%)	
**Donor ABO**			0.240
A	598 (34.7%)	422 (37.6%)	
Ab	29 (1.7%)	23 (2.0%)	
B	179 (10.4%)	99 (8.8%)	
O	918 (53.2%)	578 (51.5%)	
**Donor PHS increased risk**	659 (46.5%)	878 (78.3%)	<0.001
**Donor clinical infection**	789 (45.8%)	812 (72.4%)	<0.001
**Donor mechanism of death**			<0.001
Drug intoxication	241 (14.0%)	484 (43.1%)	
Asphyxiation	42 (2.4%)	42 (3.7%)	
Cardiovascular	167 (9.7%)	159 (14.2%)	
Gunshot wound	167 (9.7%)	55 (4.9%)	
Blunt injury	337 (19.5%)	139 (12.4%)	
ICH/stroke	702 (40.7%)	204 (18.2%)	
Death from natural causes	26 (1.5%)	13 (1.2%)	
None of the above	28 (1.6%)	19 (1.7%)	
**Liver donor risk index**	1.4 (1.1–1.6)	1.2 (1.1–1.5)	<0.001
**Cold ischemic time (hours)**	6.6 (5.0–8.6)	6.0 (4.7–7.4)	<0.001
**Recipient age (years)**	55.0 (51.0–59.0)	60.0 (55.0–63.0)	<0.001
**Recipient dialysis prior**	112 (6.5%)	139 (12.4%)	<0.001
**MELD lab score at transplant**	16.0 (12.0–22.0)	18.0 (12.0–23.0)	0.0511
**Recipient HCC diagnosis (ever)**	38 (57.6%)	456 (51.6%)	0.352
**Ascites at transplant**			<0.001
Absent	339 (21.0%)	341 (30.4%)	
Slight	852 (52.7%)	516 (46.0%)	
Moderate	417 (25.8%)	265 (23.6%)	
**Encephalopathy at transplant**			<0.001
None	536 (33.1%)	501 (44.7%)	
1–2	934 (57.8%)	549 (48.9%)	
3–4	138 (8.5%)	72 (6.4%)	
N/A	9 (0.6%)	0 (0.0%)	

MELD, Model for End-Stage Liver Disease; HCC, hepatocellular carcinoma.

## Supplementary Materials

The following are available online at http://www.mdpi.com/2079-9721/6/3/62/s1, Table S1: Demographics and clinical characteristics of HCV seropositive donors for kidney transplants.

## Abbreviations

DAA	direct-acting antiviral
HCV	hepatitis C virus
ESRD	end-stage renal disease
OPTN/UNOS	Organ Procurement Transplant Network/United Network for Organ Sharing
SVR	sustained virological response
KDPI	Kidney Donor Profile Index
MELD	Model for End-Stage Liver Disease
NAT	Nucleic Acid Testing
