# Supplementary material for: Underutilization of Hepatitis C Virus Seropositive Donor Kidneys in the United States in the Current Opioid Epidemic and Direct-Acting Antiviral Era
Source: Diseases. 2018 Jul 10;6(3):62. doi: 10.3390/diseases6030062 (PMC6165210; doi:10.3390/diseases6030062)
Supplement: Supplementary file 1 [file diseases-06-00062-s001.pdf]

Underutilization of Hepatitis C Virus Seropositive Donor Kidneys in the United States in the Current Opioid Epidemic and Direct-Acting Antiviral Era

Supplementary Materials

Table S1. Demographics and clinical characteristics of HCV seropositive donors for kidney transplants.

	HCV seropositive donor	Non-HCV Donor	P value
**Recipient Median Age (IQR)**	59 (55 - 63)	56 (45 - 64)	<0.0001
**Recipient Gender**			<0.0001
Male	1621 (80.2)	39147 (59.9)	
**Recipient Ethnicity**			<0.0001
White	498 (24.7)	26757 (40.9)	
Black	1259 (62.3)	21375 (32.7)	
Hispanic	198 (9.8)	11214 (17.2)	
Other	65 (3.2)	6000 (9.2)	
**Donor Ethnicity**			<0.0001
White	1655 (81.9)	44154 (67.6)	
Black	151 (7.5)	9328 (14.3)	
Hispanic	193 (9.6)	8944 (13.7)	
Other	21 (1)	2920 (4.5)	
**KDPI**	0.52 (0.39 - 0.69)	0.48 (0.25 - 0.71)	<0.0001
**Donor CDC HIV Risk**	1298 (64.3)	9858 (15.1)	<0.0001
**Donor gender**			0.003
Male	1282 (63.5)	39328 (60.2)	
**Donor Mechanism of death**			<0.0001
Asphyxiation	120 (5.9)	3465 (5.3)	
Blunt injury	381 (18.9)	15068 (23.1)	
Cardiovascular	212 (10.5)	9775 (15)	
Death from natural causes	27 (1.3)	1805 (2.8)	
Drowning	3 (0.1)	882 (1.3)	
Drug intoxication	665 (32.9)	4990 (7.6)	
Electrical	0 (0)	50 (0.1)	
Gunshot wound	206 (10.2)	6165 (9.4)	
ICH/stroke	358 (17.7)	20699 (31.7)	
Seizure	11 (0.5)	635 (1)	
Stab	5 (0.2)	153 (0.2)	
None of the above	32 (1.6)	1625 (2.5)	
Not Reported	0 (0)	2 (0)	
**Donor Age (median, IQR)**	33 (27 - 44)	40 (26 - 52)	<0.0001
**Donor Cancer**	44 (2.2)	2028 (3.1)	0.0177
